# Genetic Association Between *CDKN1B* rs2066827 Polymorphism and Susceptibility to Cancer

**DOI:** 10.1097/MD.0000000000001217

**Published:** 2015-11-20

**Authors:** Yongchao Lu, Kejian Gao, Miao Zhang, Aiyan Zhou, Xiaoming Zhou, Zhongan Guan, Xuewen Shi, Shujian Ge

**Affiliations:** From the Department of Traditional Chinese Medicine, Shandong Provincial Hospital Affiliated to Shandong University, Jinan, China (YL, AZ, ZG, XS); Department of Anorectal Surgery, Central Hospital of Jinan City, Jinan, China (KG); Department of Clinical Laboratory, Shandong Provincial Hospital Affiliated to Shandong University, Jinan, China (MZ); and Department of Science and Education, Shandong Provincial Hospital Affiliated to Shandong University, Jinan, China (XZ, SG).

## Abstract

Much attention has been directed to the association between cancer risk and rs2066827 polymorphism of the CDKN1B gene. However, the results are indefinitive and inconclusive. This study was devised to evaluate the hypothesis that rs2066827 polymorphism is associated with the risk of cancer.

Computer-based databases (EMBASE, PubMed, and CNKI) were used to seek all case–control studies evaluating rs2066827 polymorphism and susceptibility to cancer. The genetic risk was assessed by calculating pooled odds ratio (OR) with its corresponding 95% confidence interval (CI). Fixed-effects pooled ORs were calculated by the Mantel–Haenszel method (*P*_h_ > 0.05), and random-effects pooled ORs were estimated by the DerSimonian–Laird method (*P*_h_ < 0.05).

Data on rs2066827 polymorphism and cancer risk were available for 9038 cancer cases and 11,596 controls participating in 17 studies. Carriage of a TG genotype was associated with a minor but significant decrease in the risk of cancer (pooled OR 0.92, 95% CI: 0.86–0.99; model, TG vs. TT). We observed a moderately decreased risk of ovarian cancer based on 1829 cases and 2868 controls (pooled OR 0.85, 95% CI: 0.74–0.97; model, TG vs. TT). A slightly deceased risk of cancer was also indicated in Caucasians consisting of 6707 cases and 8279 controls (pooled OR 0.91, 95% CI: 0.85–0.98; model, TG vs. TT).

These data suggest that carriage of a TG genotype at rs2066827 polymorphism may be associated with decreased susceptibility to cancer, ovarian cancer in particular.

## INTRODUCTION

Cyclin-dependent kinases (CDKs) and CDK inhibitors (CKIs) are important regulators of cell cycle progression.^1,2^ Impaired CKI function results in indisciplinable proliferative activity. Uncontrolled cell proliferation and damage to genes involved in regulating the cell cycle are known causes of human carcinogenesis.^3^ Cyclin E forms a complex with CDK2. Cyclin E/CDK2 genes are master mediators of the cell cycle progression in the G1 phase and of the G1-S phase transition. In cancerous tissues and cancer cell lines, cyclin E can be proteolytically separated into low molecular weight (LMW) isoforms which are biologically functional. Overexpressed LMW increases cell proliferation rate and deregulates the cell cycle of mammary epithelial cells.^4^

p27 protein is encoded by the cyclin-dependent kinase inhibitor 1B (*CDKN1B*) gene mapped to chromosome 12p13. The cell cycle inhibitor protein hampers enzymatic activation of cyclin E/CDK2 complexes and induces cell cycle arrest at G1 phase.^5^ Lower expression of cyclin-dependent kinase inhibitor CDKN1B related to tumorigenesis and advanced clinical stage has been identified in multiple types of cancer, such as cancers of prostate, gastric, laryngeal, colorectal, and breast.^6–10^

Single nucleotide polymorphisms (SNPs) of the *CDKN1B* gene have been linked to reduced transcription rate and lower protein CDKN1B levels.^11^ There lies an SNP (T to G) at codon 109 that causes an amino acid change from valine to glycine (T326G, V109G, rs2066827).^12^ The common SNP may affect the normal function of *CDKN1B* by altering its transcription efficiency and protein levels, leading to cell cycle dysregulation and cancer cell behavior.^13^ A series of epidemiological studies have demonstrated increased genetic susceptibility to cancer ascribed to rs2066827 polymorphism.^11,14,15^ However, several recent studies failed to replicate this finding. We therefore performed a meta-analysis incorporating 17 studies with 9038 cancer cases and 11,596 controls to determine the association between cancer risk and rs2066827 polymorphism.

## MATERIALS AND METHODS

### Search Strategy

Human case–control studies estimating the risk of cancer associated with rs2066827 polymorphism were identified by searching EMBASE, PubMed, and CNKI databases. We used the following keywords: “cancer,” “polymorphism,” “*CDKN1B*,” “rs2066827,” and their synonyms (*p27*, T326G, V109G, variant, carcinoma) in combination, without language restrictions. Reference lists of all relevant review articles, meta-analyses, and human case–control studies addressing the association of interest were examined to obtain additional usable data. The study was supported by the Research Ethics Committee of Shandong Provincial Hospital Affiliated to Shandong University.

### Inclusion Criteria and Exclusion Criteria

Genetic association studies satisfying all of the following criteria were included in this meta-analysis: a case–control study published in a peer-reviewed journal, examined the association of rs2066827 polymorphism with cancer, reported the genotype frequencies of rs2066827 polymorphism in cases and controls in detail, and genotype frequencies in controls must be in agreement with Hardy–Weinberg equilibrium (HWE). The studies were excluded if published as an abstract with insufficient genotype data, designed as a case-case or case-only study, and comments, editorials, letters, or systematic reviews. When several articles were conducted based on the same patients, the more informative article with a larger sample size was selected for further analysis.

### Data Extraction

Data were extracted on first author, year of publication, country of study, ethnicity of each study population, selection and characteristics of cases and controls, genotyped cases and controls, cancer type, method used for genotyping, frequencies of genotypes, and results of HWE test. Data extraction was done separately by 2 of the investigators. Discrepancies were resolved by consensus involving a senior investigator.

### Statistical Analysis

Odds ratios (ORs) with the corresponding 95% confidence intervals (CIs) were calculated to assess cancer risk. Pooled ORs were estimated for 3 genetic models (GG + TG vs. TT, G vs. T, TG vs. TT) using the fixed-effects or the random-effects model. Heterogeneity across the studies was measured by the χ^2^-based Q-test, with *P* < 0.05 being considered significant. The Mantel–Haenszel method was used to calculate fixed-effects pooled ORs,^16^ and the DerSimonian–Laird method was chosen to calculate random-effects pooled ORs.^17^ Subgroup analyses according to ethnicity (Asian, Caucasian, African) and cancer type (breast cancer, ovarian cancer, and others when the cancer was investigated in less than 3 papers) were preformed to detect the potential association for each subgroup.

Sensitivity analyses were performed to evaluate influence of the individual studies on the pooled ORs. Deviation from HWE in the controls was checked by the goodness-of-fit χ^2^ test. Publication bias was estimated by inspection of funnel plot asymmetry and by calculation of Egger's linear regression method intercept.^18^ Statistical data were analyzed using STATA software package (v.12.0). The significance threshold was set at *P* < 0.05.

## RESULTS

### Characteristics of the Studies

**FIGURE 1:**
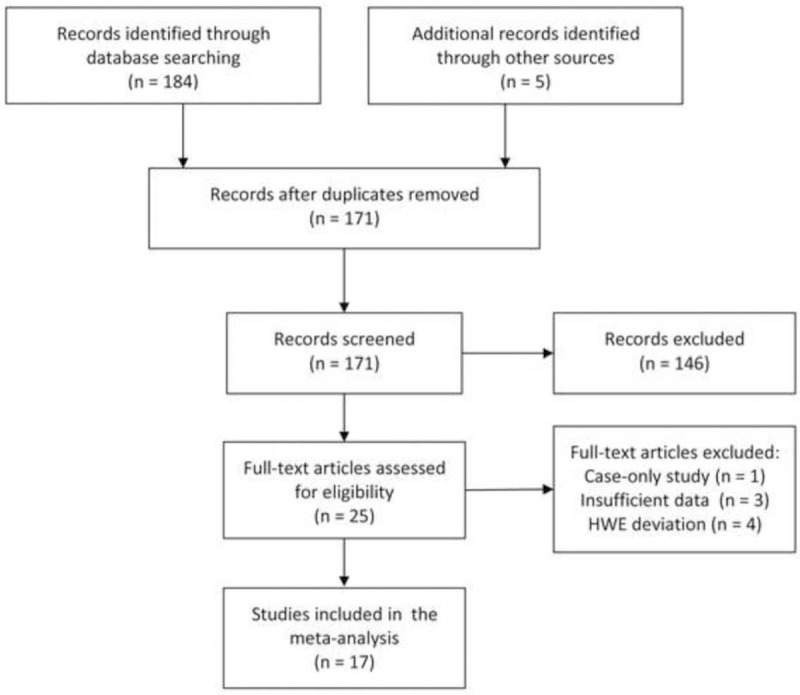
Flow chart shows study selection process.

The flow diagram of study exclusion and inclusion with specific reasons is shown in Figure 1. We identified a total of 189 records using search strategy. Among these, 25 articles appeared to be potentially eligible for inclusion and were retrieved in full texts. After full-text review, 8 articles were excluded due to case-only design (n = 1), HWE deviation (n = 4), and no detailed genotyping data (n = 3). Therefore, 17 studies were finally included in this meta-analysis.^19–35^ As shown in Table 1, 9 studies were conducted on Caucasians, 7 on Asians, and 1 on Africans. We identified 4 studies for breast cancer, 3 for ovarian cancer studies, 2 for prostate cancer, 2 for thyroid carcinoma, 1 for hepatocellular carcinoma, 1 for esophageal squamous cell carcinoma (ESCC), 1 for lung cancer, 1 for melanoma, 1 for squamous cell carcinoma of the head and neck (SCCHN), and 1 for pancreatic cancer. Regarding the choice of genotyping method, polymerase chain reaction-restriction fragment length polymorphism (PCR-RFLP) was used in most studies (n = 10).

**TABLE 1:**
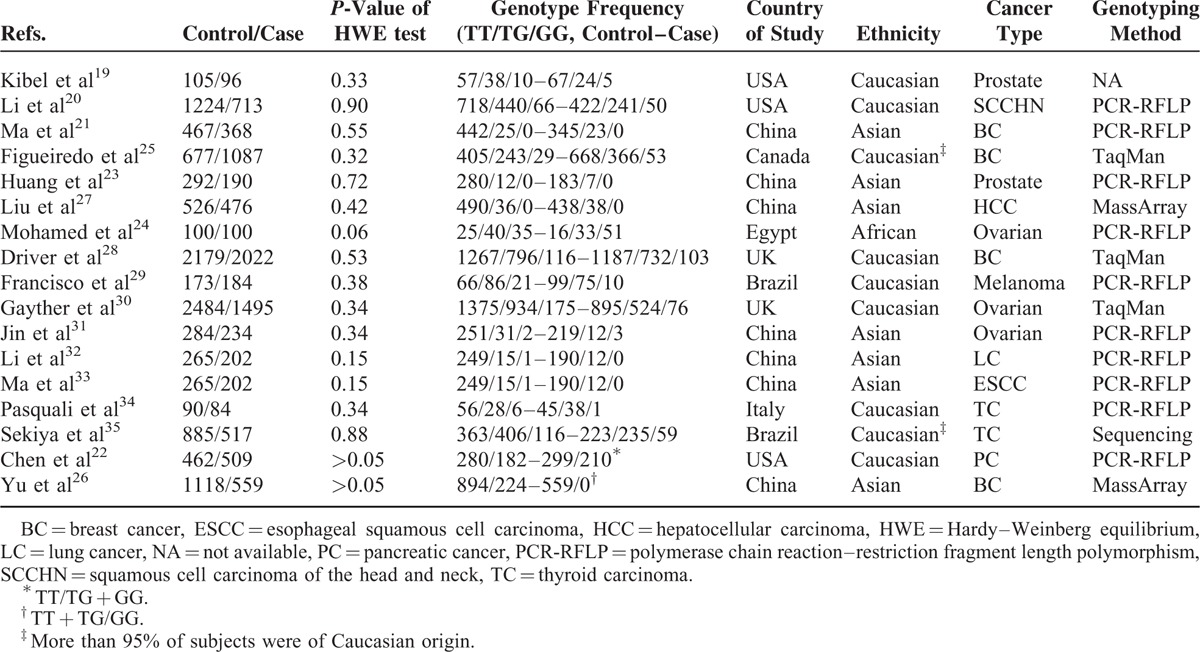
Main Characteristics of All Studies Included in the Meta-Analysis

### Meta-Analysis

**FIGURE 2:**
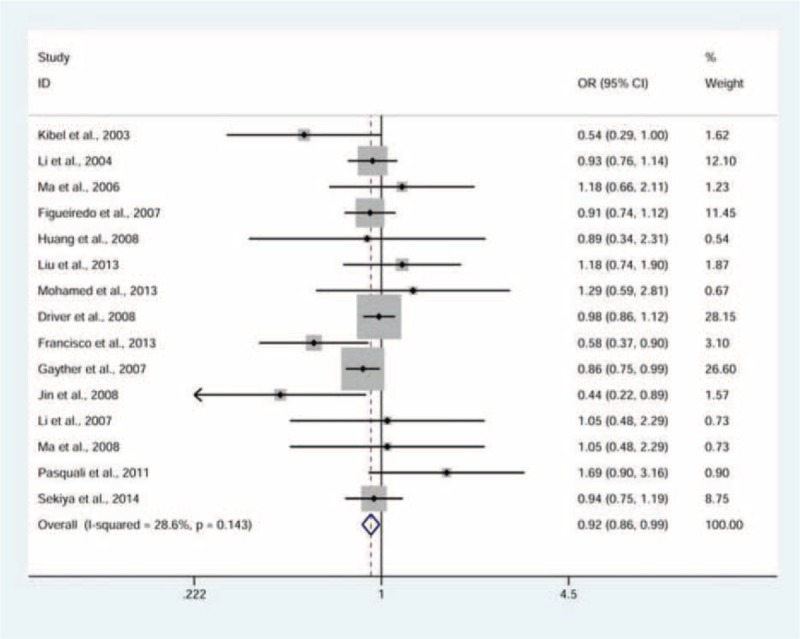
Forest plot for the association between the rs2066827 polymorphism and cancer risk (TG vs. TT model). Pooled odds ratio (OR) and 95% confidence interval (CI) have been appropriately derived from the fixed-effects model.

**FIGURE 3:**
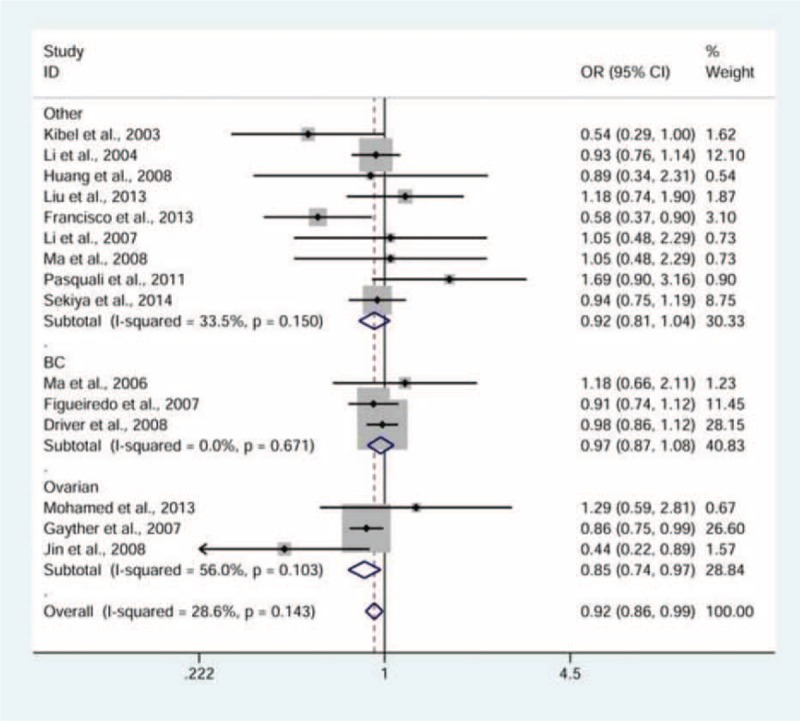
Forest plot for the association between the rs2066827 polymorphism and cancer risk by cancer type (TG vs. TT model). Pooled odds ratio (OR) and 95% confidence interval (CI) have been appropriately derived from the fixed-effects model.

There was a significant association observed between rs2066827 polymorphism and cancer risk (Table 2). The TG genotype carriers, compared to the TT genotype carriers, were less likely to develop cancer (fixed-effects pooled OR 0.92, 95% CI: 0.86–0.99; model, TG vs. TT, Figure 2). In the subgroup analyses by cancer type, a moderate decrease in the risk of ovarian cancer was seen among carriers with a TG genotype (fixed-effects pooled OR 0.85, 95% CI: 0.74–0.97; model, TG vs. TT, Figure 3). We finally stratified the studies by ethnicity, and found a slightly decreased risk of cancer associated with the TG genotype in Caucasians (fixed-effects pooled OR 0.91, 95% CI: 0.85–0.98; model, TG vs. TT, Table 2).

**TABLE 2:**
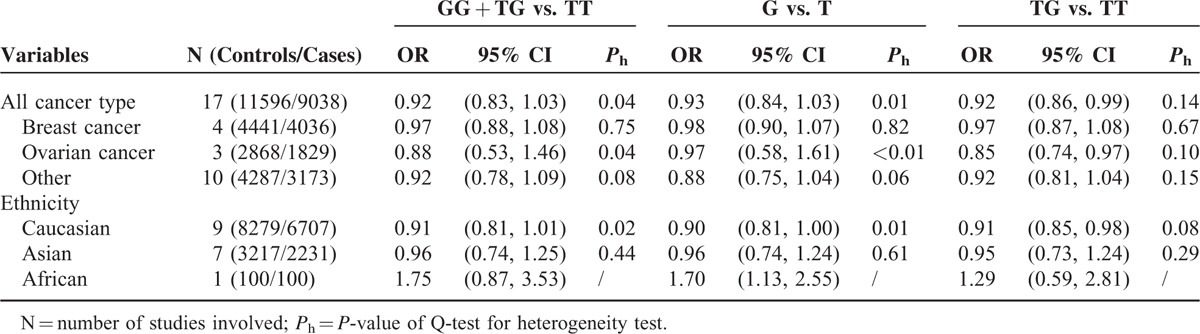
Main Results of Pooled ORs and Stratification Analysis of rs2066827 Polymorphism on Cancer Risk in the Meta-Analysis

### Heterogeneity Test and Sensitivity Analysis

We detected significant between-study heterogeneity in the analyses using GG + TG vs. TT, and G vs. T models (*P* < 0.05, Table 2). We performed sensitivity analyses by sequentially omitting the studies included (one omitted each time). Two studies, by Mohamed et al^24^ and Francisco et al^29^, respectively, were identified as outliers. The results in GG + TG versus TT, and G versus T models were highly homogeneous when the outliers were excluded (available on request). But we noted that the exclusion resulted in significant alternations in the pooled ORs (available on request).

### Publication Bias

**FIGURE 4:**
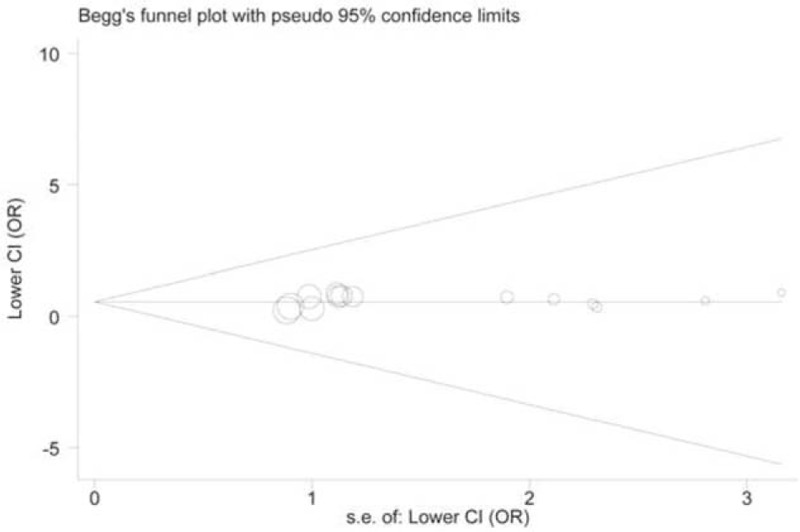
Funnel plot of publication bias analysis for the associations between the rs2066827 polymorphism and cancer risk (TG vs. TT).

Publication bias is a potential problem when performing a meta-analysis. To determine the possible bias, we used Begg funnel plots and Egger test simultaneously. Both tests showed no evidence of significant bias (*P*_Begg_ = 0.73, *P*_Egger_ = 0.43, model: TG vs. TT) (Figure 4).

## DISCUSSION

Cell cycle progression is mediated by cyclins and cyclin-dependent kinase complexes. The CDKN1B mammalian cell cycle protein has been identified as an inhibitor of cyclin-dependent kinases.^36^ Lack of CDKN1B is thought to be an important mechanism underlying carcinogenesis.^37–40^

Due to the influence of nonsynonymous SNPs on the amino acid sequence of protein, the role of a common SNP at codon 109 that encodes a nonsynonymous amino acid change of CDKN1B in the pathogenesis of human cancer has received widespread attention.^19–23^ Nevertheless, these genetic association studies produced highly controversial findings, failing to facilitate a full understanding of the mechanisms that underlie cancer. For instance, Li et al^20^ designed a hospital-based case–control study incorporating 713 patients with SCCHN and 1224 cancer-free control subjects of Caucasian origin, demonstrating no evidence for a major role of rs2066827 in the etiology of SCCHN. In a subsequent case–control study of 368 breast cancer patients and 467 controls of Asian origin, Ma et al^21^ did not observed a significantly increased or decreased risk of breast cancer. It is especially interesting that Figueiredo et al^25^ identified a modest protective effect on breast cancer associated with the G allele in a population-based study of 1115 cases and 710 controls of Caucasian ancestry. There are several possible causes related to the inconsistency. The first cause is the sampling difference. A study with a larger number is known to be more prone to reveal the true SNP–cancer association. The second reason may be due to the heterogeneous populations and ethnic variance. Possible consequences resulting from various genetic backgrounds include allele frequency variance, different gene–gene interactions and gene–environment interactions. The third cause might be the different cancer types. Human cancers at various sites may vary widely in terms of the etiology of pathology. In this study, we carried out a meta-analysis with an aim to examine the association between rs2066827 polymorphism and cancer risk.

In our meta-analysis of 9038 cancer cases and 11,596 controls participating in 17 studies, we demonstrated a weak protective effect on cancer in relation to the TG genotype. We also observed a moderate protective effect on ovarian cancer and a slight protective effect in Caucasians after stratification by cancer type and ethnicity, respectively. The most likely reason for absence of an association in Asians may relate to the significant difference in minor allele frequency (G). The frequency in the Asian populations was 3.4%, which was significantly lower compared to Caucasians (25.9%, *P* < 0.05). Another possible explanation is the wide disparity of total number (8279 cases and 6707 controls of Caucasian origin, 3217 cases and 2231 controls of Asian origin).

In a previous meta-analysis where a total of 8 studies with 3591 cases and 3799 controls were included, Wei et al^41^ failed to demonstrate any evidence in support of an association between rs2066827 polymorphism and cancer. In this published analysis, Wei et al included a study of 398 breast cancer cases and 372 controls,^42^ which was later updated by Figueiredo et al in a larger number.^25^ The results thus may be affected due to the overlapped data. Unlike the earlier meta-analysis, our analysis restricted to studies without HWE deviation indicated significant protective effects on cancer based on 13,244 additional subjects (5447 cases and 7797 controls). In addition, we found a novel association for ovarian cancer and Caucasians after excluding the overlapped data.

There are some strengths and limitations in our study. An important strength is the markedly expanded sample size which helped to identify some findings not suggested in the previous meta-analysis. Further, we included all studies with usable data without considering language. The exclusion of papers for linguistic reasons may lead to biased results.^43^ However, the current sample was too small to detect the possible effects for multiple cancer types and did not allow further stratification analyses by ethnicity for ovarian cancer and breast cancer. Thus, sample insufficiency is one of the limitations. Second, significant heterogeneity presented in some analyses. Exclusion of the identified outliers caused obvious changes in the risk estimates, suggesting that heterogeneity should be considered in interpreting some results. Finally, effects of common confounding factors, including age, gender, tobacco smoking and alcohol consumption, were not assessed in the present study because of data insufficiency.

In summary, our meta-analysis demonstrated some evidence that the rs2066827 polymorphism of the CDKN1B gene may protect against the development of human cancer, especially ovarian cancer and in Caucasians. Additional work with a larger number is recommended to better understand the potential functional role of this nonsynonymous polymorphism in distinct ethnic populations and various cancers.
